# Continued increases in blood pressure over two decades in Samoa (1991–2013); around one-third of the increase explained by rising obesity levels

**DOI:** 10.1186/s12889-018-6016-2

**Published:** 2018-09-15

**Authors:** Christine Linhart, Take Naseri, Sophia Lin, Richard Taylor, Stephen Morrell, Stephen T. McGarvey, Dianna J. Magliano, Paul Zimmet

**Affiliations:** 1 Public and International Health, School of Public Health and Community Medicine, University of New South Wales, Sydney, Australia; 2 Ministry of Health, Apia, Samoa; 3 International Health Institute, Department of Epidemiology, Brown University School of Public Health, Providence, USA; 4 Baker IDI Heart & Diabetes Institute, Melbourne, Australia

**Keywords:** Samoa, Hypertension, Blood pressure, Risk factors, Obesity

## Abstract

**Background:**

To analyse trends over the period 1991–2013 in systolic blood pressure (SBP), diastolic blood pressure (DBP) and the prevalence of hypertension in adults aged 25–64 years in Samoa; and to assess the contribution of rising obesity levels to period trends.

**Methods:**

Unit record data from seven population-based surveys (*n* = 10,881) conducted between 1991 and 2013 were included for analysis. Surveys were adjusted to the nearest previous census to improve national representativeness. Hypertension was defined as SBP ≥140 mmHg and/or DBP ≥90 mmHg and/or on medication for hypertension. Obesity was measured by body mass index (BMI). Poisson, linear and meta-regression were used to assess period trends.

**Results:**

Over 1991–2013 mean SBP and DBP (mmHg), and the prevalence of hypertension (%) increased in both sexes. Increases in hypertension were: from 18.3 to 33.9% (*p* < 0.001) in men (mean BP from 122/74 to 132/78); and from 14.3 to 26.4% (*p* < 0.001) in women (mean BP from 118/73 to 126/78). The estimate of the age-adjusted mean SBP and DBP over 1991–2013, and the relative risk for hypertension in 2013 compared to 1991, were attenuated after adjusting for BMI: by 22% (men) and 32% (women) for mean SBP; 37% (men) and 32% (women) for mean DBP; and 19% in both sexes for hypertension.

**Conclusions:**

Significant increases have occurred in SBP/DBP and hypertension prevalence in both sexes in Samoa during 1991–2013, which would contribute significantly to premature mortality from cardiovascular disease. Obesity accounts for around one-third of the rising trend in blood pressure in the Samoan population. Strengthening of population control of hypertension through reduction in obesity and salt intake, and case detection and treatment through primary care, is required to reduce premature mortality from cardiovascular disease in Samoa.

## Background

Samoa is an island nation in the South Pacific region with a population of 196,000 at the 2016 Census [1]. Since the mid-twentieth Century mortality levels have declined in Samoa, particularly among children under-five years, and the leading causes of death have changed from infections and under-nutrition to cardiovascular disease (CVD), cancer and other non-communicable diseases (NCD) [2]. Long term trends in risk factors for NCDs have been partly documented in Samoa by examination of increasing obesity and type 2 diabetes mellitus (T2DM) prevalences from 1978 to 2013 [3], and steady decline in tobacco smoking over the same period, although among Samoan men the prevalence of smoking was more than three times higher than in Australia and New Zealand in 2013 [4]. Another important risk factor for CVD is hypertension. Over the past three decades several surveys measuring blood pressure (BP) have been conducted in Samoa, with varied methodologies and definitions of hypertension. Through access to unit record data the present study applies a standardised definition of hypertension and methodology for analysis to establish population trends in hypertension prevalence, systolic blood pressure (SBP) and diastolic blood pressure (DBP) by sex in adults aged 25–64 years from seven population-based cross-sectional surveys (*n* = 10,881) conducted in Samoa over 23-years (1991–2013). The effect of increased obesity in Samoa over 1991–2013 [3] on period trends in BP is also investigated.

Reduction in CVD mortality can be achieved through risk factor reduction in individuals and populations, including interventions targeting population groups identified at high-risk. Reductions in premature adult mortality from decline in CVD occurred from the 1970s in Northern Europe, North America, Australia, and New Zealand [5], and led to significant increases in life expectancy in these populations. In Australia and New Zealand 80% of the CVD mortality decline has been associated with declines in population risk factors, including hypertension [6, 7]. The Samoa Ministry of Health (MoH) has highlighted that it is imperative to identify the impact or outcome of the NCD interventions that have been implemented in Samoa [8]; however, nationally representative period trends in hypertension from multiple comparable cross-sectional surveys over several decades have not previously been estimated. The present study establishes population trends in hypertension to aid evaluation of the effects of prevention and control interventions for BP reduction over the past 23 years in Samoa. This includes the Healthy Islands Health Promotion Project initiated in 1995 which identified hypertension, diabetes and respiratory problems as a priority focus for health promotion in Samoa [9], and under this project remained a priority focus over the next two decades [10]. Population sub-groups at higher risk of CVD because of increasing hypertension prevalence, SBP and DBP are also identified to assist policy and planning of targeted prevention and control interventions.

## Methods

### Survey selection and sample adjustments

Surveys measuring BP in adults aged 25–64 years in Samoa during 1950–2015 were included in this analysis if they were nationally representative at the time of the survey, or could be adjusted to the nearest previous census (by Division of residency, age and sex). Over the past 20 years there has been minimal change in the Samoan population structure due to a counterbalance between high out-migration and high total fertility rates of around 4.7 per woman during this period [11, 12]. During 1991–2011, population growth has remained relatively stable at below 1%; adults aged 55–64 years have constituted 14–15% of the total population aged 25–64 years; and the urban population has remained 20–21% of the total population [12].

Surveys included in analyses are: the 1991 Non-Communicable Disease Risk Factor (NCDRF) Survey (*n* = 1524) [13]; the Samoan Adiposity and Cardiovascular Disease Risk Factor (SACRF) longitudinal study in 1991 (*n* = 730) and 1995 (*n* = 531) [14, 15]; the 2002 Samoa STEPS survey (*n* = 2543) [16]; the Samoan Family Study of Overweight and Diabetes (SFSOD) survey conducted in 2003 (*n* = 683) [17]; the 2010 Samoan Genome-Wide Association Study (*n* = 3422) [18]; and the 2013 Samoa STEPS survey (*n* = 1448) [19]. Primary unit record data from each of the seven surveys was supplied to the authors in 2015 by researchers or organisations who conducted the surveys, and a combined unit record data set was assembled (*n* = 10,881). The 1978 BP survey by Zimmet et al. [20] was not included in the current analysis because of irreconcilable differences in methodology used to obtain BP measurements.

The 1991 and 1995 SACRF surveys were originally designed as T2DM incidence studies which excluded participants with known T2DM or hypertension at the time of the 1991 survey. To adjust for under-enumeration of known hypertension, a ratio of known:new hypertensive cases was derived by 20-year age-group and by Division of residence for each sex, from the 1991 NCDRF, and applied to the 1991 SACRF, as per the method described by Lin et al. [3]. Hypertension prevalence was calculated from these aggregate data following the method described by Armitage et al. [21] which also produces a standard error (SE). The same ratio was applied to the 1995 SACRF, except that in this survey known cases of T2DM and hypertension (those found to be new T2DM or hypertensive in the 1991 SACRF) were excluded before an adjusted hypertension prevalence was estimated. This is the same adjustment method used by Lin et al. [3] to investigate period trends in T2DM in Samoa over 1978–2013.

### Data collection

The 1991 NCDRF survey and the 1991 and 1995 SACRF surveys measured BP manually with random zero mercury sphygmomanometers; all other surveys used digital aneroid automatic BP machines. Both methods eliminate observer bias from subsequent measurements. In all surveys SBP was recorded at the level of appearance of sound and DBP at the level of its disappearance (phase 5). All surveys took two BP measurements from participants who were seated for 10 min, with a third BP reading taken if the difference between measurements was ≥10 mmHg. The average of the last two readings was used for analysis in all surveys. Hypertension was defined as SBP ≥140 mmHg and/or DBP ≥90 mmHg and/or taking medication for hypertension (self-reported) [22]. Pregnant women were excluded from analyses.

### Demographic adjustment and trend analyses

In order to improve national representativeness and minimise selection bias, each survey was adjusted to the most recent previous census by age group, sex, and Division of residency, using case weights derived from the ratio of the population proportions from the census and the survey for each stratum. This is the same methodology used in the WHO STEPS surveys [19]. Each survey was also age standardized to the 2011 census population to determine the effect of changes in the age structure of the population on BP means and prevalences during 1991–2013. Period trends in hypertension were analysed using random effects meta-regression, and period trends in mean SBP and DBP were analysed using linear regression. A weighted average of hypertension prevalence was calculated from the 1991 NCDRF survey and 1991 SACRF survey in order to report the hypertension prevalence for 1991.

Effects of population changes in body mass index (BMI) on hypertension prevalence and mean BP over 1991–2013 were investigated by modelling period alone (as year of each survey), with the beta estimate for each incremental year raised to the power of 23 to estimate the prevalence ratio (PR) for 2013 compared to 1991 (using Poisson regression). The contribution of age and BMI (as continuous variables) to the change in the regression estimate for period was assessed when age and BMI were successively included in the models. The modelled hypertension prevalence in 2013 (by sex) was then calculated by multiplying the baseline prevalence in 1991 by: (1) the PR for period; (2) the PR for period and age; and (3) the PR for period, age and BMI. Increases in mean SBP and DBP over the period were analysed in a similar fashion using linear regression. Analysis of effects of BMI on mean BP and hypertension was restricted to surveys that could be analysed as individual unit record data (1991 and 1995 SCARF surveys not included). SAS version 9.4 was used for analysis (SAS Institute Inc., Cary, NC, USA).

## Results

### Trends in mean SBP and DBP

**Fig. 1:**
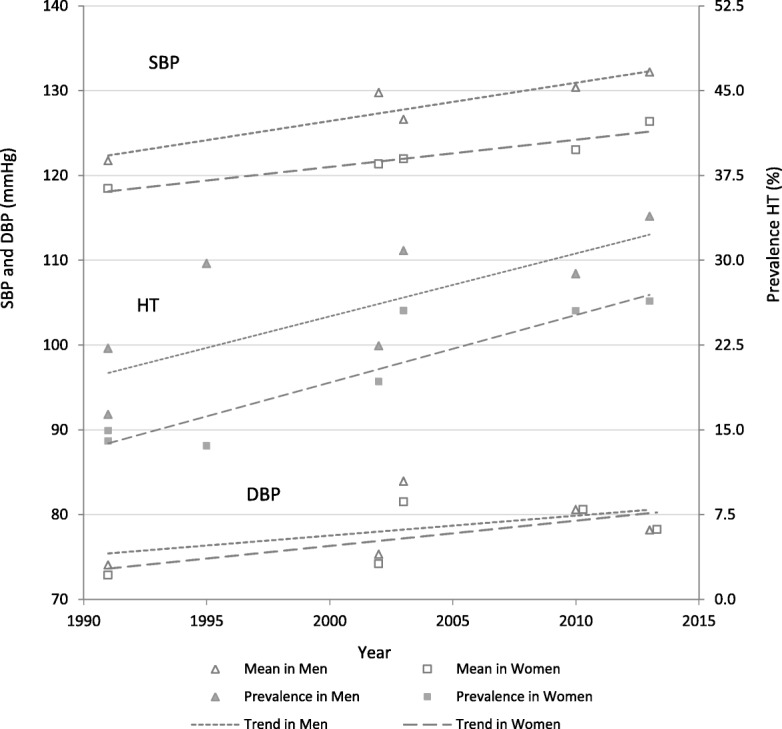
Hypertension prevalence and mean systolic/diastolic blood pressure, by sex, Samoa, 1991–2013^+.^ SBP (systolic blood pressure); DBP (diastolic blood pressure); HT (hypertension = SBP ≥140 and/or DBP ≥90 mmHg and/or taking medication for HT); +data adjusted to the most recent previous census for age group and Division of residency by sex. Mean BP not available for 1991b and 1995 surveys because of exclusions (see methods)

Trend increases in mean SBP and DBP from 1991 to 2013 in both sexes are statistically significant (*P* < 0.001) (Table 1 and Fig. 1). Attenuation of the regression estimate for period trend on mean SBP and DBP after age-adjustment is 6% in men and 13% in women for SBP (*p* < 0.001); and 9% in men and 5% in women for DBP (*p* < 0.001), with the regression estimates for period on SBP and DBP remaining statistically significant (*P* < 0.001) for both sexes (Table 2).

**Table 1:** Hypertension prevalence by sex, Samoa, 1991–2013^

Year	N	HT (%)	Systolic BP	Diastolic BP
Men				
1991a	691	16.4 (13.6–19.1)	121.7 (120.5–123.0)	74.1 (73.2–74.9)
1991b	347	22.2 (17.2–27.2)	–	–
1995	256	29.7 (23.0–36.4)	–	–
2002	1197	22.4 (20.1–24.8)	129.8 (128.9–130.7)	75.3 (74.6–76.1)
2003	316	30.8 (25.8–35.9)	126.6 (124.8–128.5)	84.0 (82.6–85.3)
2010	1390	28.8 (26.4–31.2)	130.4 (129.6–131.2)	80.6 (80.0–81.3)
2013	581	33.9 (30.0–37.7)	132.2 (130.8–133.6)	78.2 (77.2–79.2)
*5-yr change*		+ 2.78* (2.47–2.95)	+ 2.13* (1.79–2.46)	+ 1.44* (1.17–1.70)
Women				
1991a	833	14.0 (11.6–16.4)	118.4 (117.3–119.6)	72.8 (72.1–73.6)
1991b	384	15.1 (11.1–19.1)	–	–
1995	275	14.8 (9.56–18.0)	–	–
2002	1346	19.3 (17.2–21.4)	121.4 (120.3–122.4)	74.2 (73.5–74.9)
2003	367	25.6 (21.1–30.0)	122.0 (120.1–123.8)	81.5 (80.3–82.7)
2010	2031	25.5 (23.4–27.2)	123.0 (122.2–123.8)	80.6 (80.0–81.1)
2013	867	26.4 (23.4–29.3)	126.4 (125.0–127.7)	78.3 (77.4–79.1)
*5-yr change*		+ 2.99* (2.86–3.79)	+ 1.49* (1.15–1.84)	+ 1.79* (1.56–2.02)

1991a (NCDRF survey); 1991b (SACRF survey); *p < 0.001; 95% confidence intervals in brackets after the estimate; BP (blood pressure); HT (hypertension = systolic BP ≥140 and/or diastolic BP ≥90 mmHg and/or taking medication for HT); N (number of participants in stratum); ^ data adjusted to the most recent previous census for age group and Division of residency by sex to improve representativeness; 5-yr change (represents the change in each 5 year period from meta-regression for HT and linear regression for BP means); mean BP not available for 1991b and 1995 surveys because of exclusions (see methods)

**Table 2:** Effects of age and body mass index (BMI) on period trends for hypertension prevalence and mean blood pressure by sex, Samoa, 1991–2013

Variable in the model	Effect of period on hypertension trends over 23 years (1991–2013)			
	Men		Women	
	RR (95%CI)	HT % 2013^	RR (95%CI)	HT % 2013^
1. Period	2.02 (1.64–2.49)	33.1	1.95 (1.59–2.38)	27.3
2. Period and Age	1.90 (1.55–2.34)	31.1	1.82 (1.49–2.22)	25.5
*Proportional attenuation (1–2)* ^*a*^	*6.06%*	*6.06%*	*6.70%*	*6.70%*
3. Period, Age and BMI	1.54 (1.25–1.90)	25.2	1.47 (1.21–1.80)	20.7
*Proportional attenuation (2–3)* ^*a*^	*18.9%*	*18.9%*	*18.9%*	*18.9%*
Variable in the model	Increase and mean SBP (mmHg) over 23 years (1991–2013)			
	SBP (95%CI)	SBP 2013^	SBP (95%CI)	SBP 2013^
1. Period	9.77 (8.23–11.32)	131.5	6.87 (5.27–8.47)	125.3
2. Period and Age	9.20 (7.69–10.71)	130.9	5.96 (4.51–7.41)	124.4
*Proportional attenuation (1–2)* ^*a*^	5.84%	5.84%	13.2%	13.2%
3. Period, Age and BMI	7.17 (5.72–8.63)	128.9	4.04 (2.60–5.49)	122.5
*Proportional attenuation (2–3)* ^*a*^	22.1%	22.1%	32.2%	32.2%
Variable in the model	Increase and mean DBP (mmHg) over 23 years (1991–2013)			
	DBP (95%CI)	DBP 2013^	DBP (95%CI)	DBP 2013^
1. Period	6.58 (5.37–7.79)	80.6	8.26 (7.19–9.33)	81.1
2. Period and Age	5.97 (4.81–7.13)	80.0	7.85 (6.82–8.87)	80.7
*Proportional attenuation (1–2)* ^*a*^	*9.18%*	*9.18%*	*5.0%*	*5.0%*
3. Period, Age and BMI	3.77 (2.70–4.84)	77.8	5.32 (4.35–6.29)	78.2
*Proportional attenuation (2–3)* ^*a*^	*36.9%*	*36.9%*	*32.2%*	*32.2%*

1–3: Regression models; RR [relative risk]; HT [hypertension]; SBP [systolic blood pressure]; DBP [diastolic blood pressure]; ^a^Proportional decline from previous model; ^ [predicted HT prevalence and mean SBP/DBP in 2013 derived from multiplication of the baseline for HT prevalence in 1991 by the relative risk, and addition of the baseline SBP/DBP in 1991 to the period increase]

### Trends in hypertension prevalence

There are significant trend increases (*P* < 0.001) from 1991 to 2013 in hypertension prevalence in both men and women (Table 1 and Fig. 1). After adjusting for age, the effect of period trend on hypertension prevalence is attenuated by 6.1% in men and by 6.7% in women (*p* < 0.001), with the regression estimate for period trend remaining statistically significant (*P* < 0.001) for both sexes (Table 2).

### Effect of increasing age and BMI on the period (1991–2013) effect in hypertension prevalence and mean SBP and DBP

As measured by attenuation of the period effect on hypertension prevalence and mean SBP and DBP by BMI and age, compared with age alone (i.e. the age-adjusted effect of overweight/obesity), the regression models show that BMI attenuates the period effect: by 19% in both sexes for hypertension prevalence; by 22% in men and 32% in women for mean SBP; and by 37% in men and 32% in women for mean DBP (Table2). Attenuation of the regression estimate for period on hypertension prevalence and mean SBP and DBP from BMI is significant (*P* < 0.001). The regression estimates for period remain significant (*P* < 0.001) for hypertension prevalence, SBP and DBP in both sexes after adjusting for age and BMI (Table 2). The prevalence of hypertension and mean SBP/DBP (mmHg) in 2013 predicted by period adjusted for age is 31.1% (with mean BP = 130.9/80.0) in men and 25.5% (mean BP = 124.4/80.7) in women; and after adjusting for age and BMI the period predicted hypertension prevalence in 2013 is 25.2% (and mean BP = 128.9/77.8) in men and 20.7% (mean BP = 122.5/78.2) in women (Table 2).

## Discussion

Empirical survey data for Samoan adults aged 25–64 years demonstrate a statistically significant continued increase in mean SBP and DBP, and the prevalence of hypertension, in both sexes during 1991–2013. The most affected population group is men, who had higher hypertension prevalence and mean SBP throughout 1991–2013, however, the rate of the increase in hypertension over time is similar in both sexes. The sex differential in DBP slowly diminished from a higher level in men in 1991, to reach parity with women by 2013. This is similar to findings in Fiji of a statistically significant continued increase in hypertension prevalence, SBP and DBP in both sexes of the i-Taukei and Indian populations during 1980–2011 [23]. However, the prevalence of hypertension and mean SBP and DBP remains lower in Samoa in the most recent period compared to Fiji where hypertension prevalence was 40% in men (34% in Samoa) and 35% in women (26% in Samoa) [23]. Comparison of WHO NCD STEPS surveys undertaken between 2002 and 12 in 15 Pacific Island Countries and Territories found that in most populations the prevalence of hypertension exceeded 20%, with the highest age-standardised prevalence in the Cook Islands and American Samoa at 44% in men and 32–33% in women [24].

Previous population studies of BP in Samoa dating back to the 1970s have identified an association between increasing age and increases in mean BP and hypertension prevalence in both sexes [20]. Increased BP with increasing age has also been found in other Pacific Islands, including Fiji [25], Tonga [26], and American Samoa [27, 28] and elsewhere, including Australia [29]. However, some previous studies of least modernised populations indicate no significant increase in BP with age in the Pacific, including the northern Cook Islands (Pukapuka) [30], Tokelau [31], and Wallis Island [32], and elsewhere, including Brazil and Kenya [33], highlighting that increased BP is not an inevitable consequence of ageing.

Possible limitations of the present study are that BP in Samoa increased with age in the period under investigation, and changing proportions of the population in age groups over time could confound the relationship of BP to period. To determine the extent to which this affected the results, age standardised analyses to the 2011 census were undertaken of the concatenated surveys, compared with adjustment to the nearest previous census, and regression estimates for period after adjusting for age in the regression models were established. This resulted in a small attenuation of the effect of period on the increase in hypertension prevalence in men over the period (1991–2013), with larger attenuations in women. The period effect, however, remained statistically significant in both sexes after adjusting for age in both ways.

Proximate risk factors, including obesity [34] and salt intake [35] contribute significantly to increases in BP. The prevalence of obesity in both sexes in Samoa has increased over recent decades, with higher levels in women compared with men [3]. The effect of obesity on period as a predictor of hypertension prevalence and mean SBP and DBP was assessed by adding BMI to the models including age and period as predictors. Attenuation of the period effect on hypertension prevalence and mean DBP by BMI is similar in both sexes, whilst attenuation of the period effect on mean SBP is greater in women. The largest attenuation of the period effect by BMI, after age adjustment, was on mean DBP, where BMI accounts for around one-third of the period increase in both sexes. This is similar to findings in Fiji over 1980–2011, where the most substantial impact of BMI on period trends in both sexes and ethnicities (i-Taukei and Fijians of Indian descent) is on DBP [23]. DBP, as a measure of peripheral resistance, is the physiological lesion in essential hypertension. SBP is a better predictor of coronary artery disease (atherosclerosis) because it is partly a consequence of aortic stiffening due to similar pathology. For the period increase in SBP in Samoa over 1991–2013, BMI accounts for just under one-third of the trend increase in women, and one-quarter in men; and for hypertension prevalence it accounts for around one-quarter of the increase in both sexes.

Salt intake has not been adequately measured in population surveys in Samoa to allow accurate estimation of trends over time. The 1991 NCDRF survey estimated sodium intake through 24-h dietary recall method (*n* = 491) and reported a positive association between years of education and sodium intake in both sexes; and significantly higher sodium intake in the administrative/clerical occupational category compared to the agriculture/labour category [36]. This is similar to findings in other Pacific islands during the 1980s including Fiji, Niue and Kiribati, where more modernised populations had higher urine sodium output than more rural areas and outer islands based to a significant extent on a subsistence economy [37]. From the 2013 STEPS survey a sub-sample was selected (*n* = 500 randomly selected; 58.5% participation rate; *n* = 293 used for analysis) to assess salt intake [38]. The mean population 24-h urine excretion of salt was 7.63 g (SE 0.27) for men and 6.39 g (SE 0.14) for women; with salt intake increasing with BMI (both sexes combined, *p* < 0.001). These levels are lower than modelled average intake levels for other low- and middle-income countries [39], however, 70% of the sample had urinary salt excretion of > 5 g/day (2 g sodium) [19, 38], above WHO guidelines [40]. These salt intake guidelines are primarily based on evidence of the positive relationship between salt intake and blood pressure established by the INTERSALT study [35]; with evidence for an association with all-cause mortality and CVD less clear [40].

## Conclusions

The present study is the first to identify trends in mean SBP and DBP, and hypertension prevalence in Samoa by sex from large cross-sectional surveys over a 23-year period (1991–2013) using a standardised definition of hypertension and methodology for analysis. The findings indicate that preventive approaches relating to obesity from energy intake and inadequate physical activity, reduction in salt intake, and identification and treatment of cases of hypertension in the Samoan population [2] need to be strengthened since decreases in hypertension prevalence or SBP and DBP are not yet evident. Rather, trends demonstrate a continued increase over the last 23 years. Reduction in CVD risk factors, including BP, has been achieved in recent decades in populations such as Australia, associated with reduction in population risk factor levels [6]. The present study indicates that the most affected population group in Samoa are men, who had the highest levels of hypertension prevalence and mean SBP in 2013, and the greatest rates of increase in these measures during 1991–2013, leading to almost a doubling of prevalence. Although public health interventions aimed at CVD risk factor reduction are being expanded across the entire Samoan population [2], additional interventions targeting high-risk population groups, particularly men, may be necessary.

## Abbreviations

BMI: body mass index
BP: blood pressure
CVD: cardiovascular disease
DBP: diastolic blood pressure
MoH: Ministry of Health
NCD: non-communicable disease
NCDRF: Non-Communicable Disease Risk Factor
PR: prevalence ratio
SACRF: Samoan Adiposity and Cardiovascular Disease Risk Factor
SBP: systolic blood pressure
SFSOD: Samoan Family Study of Overweight and Diabetes
T2DM: type 2 diabetes mellitus
WHO: World Health Organisation
